# PSPC1 Binds to HCV IRES and Prevents Ribosomal Protein S5 Binding, Inhibiting Viral RNA Translation

**DOI:** 10.3390/v16050738

**Published:** 2024-05-07

**Authors:** Sachin Kumar Tripathi, Ashish Aneja, Teji Borgaonkar, Saumitra Das

**Affiliations:** 1 Department of Microbiology and Cell Biology, Indian Institute of Science, Bangalore 560012, Karnataka, India; 2 National Institute of Biomedical Genomics, Kalyani 741251, West Bengal, India

**Keywords:** hepatitis C virus, paraspeckles, PSPC1, IRES, ribosomal protein S5

## Abstract

Hepatitis C virus (HCV) infects the human liver, and its chronic infection is one of the major causes of Hepatocellular carcinoma. Translation of HCV RNA is mediated by an Internal Ribosome Entry Site (IRES) element located in the 5’UTR of viral RNA. Several RNA Binding proteins of the host interact with the HCV IRES and modulate its function. Here, we demonstrate that PSPC1 (Paraspeckle Component 1), an essential paraspeckle component, upon HCV infection is relocalized and interacts with HCV IRES to prevent viral RNA translation. Competition UV-crosslinking experiments showed that PSPC1 interacts explicitly with the SLIV region of the HCV IRES, which is known to play a vital role in ribosomal loading to the HCV IRES via interaction with Ribosomal protein S5 (RPS5). Partial silencing of PSPC1 increased viral RNA translation and, consequently, HCV replication, suggesting a negative regulation by PSPC1. Interestingly, the silencing of PSPC1 protein leads to an increased interaction of RPS5 at the SLIV region, leading to an overall increase in the viral RNA in polysomes. Overall, our results showed how the host counters viral infection by relocalizing nuclear protein to the cytoplasm as a survival strategy.

## 1. Introduction

The HCV genome is flanked by the 5’ and 3’ untranslated regions (UTRs), critical for both translation and replication of the viral RNA. The 5’ UTR has an Internal ribosome entry site (IRES), which is required for viral RNA translation. The IRES comprises the first 24–40 nucleotides of the core coding region and domains II, III, and IV of the 5’ UTR [1]. The propagation of HCV depends on host factors such as proteins, miRNAs, and lncRNAs [2,3,4]. It has been shown that several cellular proteins are required for HCV translation, such as Human La autoantigen (La), poly(rC)-binding protein 2 (PCBP2), and ribosomal protein S5 (RPS5) [5,6,7,8,9,10,11,12,13,14]. Apart from RNA-binding proteins, miRNAs, including miR-122, which is expressed especially in liver cells, enhance HCV translation and replication, and impact the virus’s stability [15,16,17]. Recent reports suggested that several lncRNAs regulate viral replication, translation, and release [3,18].

Paraspeckles are ribonucleoprotein bodies found in the interchromatin space of mammalian cell nuclei which are composed of long non-protein-coding RNA species, NEAT1/Men ε/β and core paraspeckle proteins which are members of the DBHS (Drosophila Behavior Human Splicing) family of proteins: P54NRB/NONO, PSPC1, and PSF/SFPQ [19]. These structures facilitate the nuclear retention of RNA, which controls the expression of specific genes in differentiated cells [20]. Paraspeckle proteins SFPQ and PSF and lncRNA NEAT1 are known to regulate several virus life cycles, such as influenza virus, HIV-1, Coxsackievirus B3, HDV, and HSV-1 [21,22,23,24,25,26]. PSPC1 has also been reported to regulate the virus life cycle of other viruses, such as through the relocalization of PSPC1 to cytoplasmic foci upon HDV infection, it increases HDV replication via interacting with the HDV genome [27]. Additionally, PSPC1 has also been shown to regulate HSV-1 replication and gene expression [25,28].

In the current study, we investigated the role of PSPC1, a paraspeckle component and an RNA binding protein, in regulating the HCV life cycle. We investigated the effect of HCV infection on the localization of PSPC1 and showed that PSPC1 interacts with the SLIV region of the HCV IRES and competes with the RPS5 protein, thereby negatively regulating viral RNA translation. Overall, our work shows the functional regulation of HCV IRES activity by PSPC1 protein. 

## 2. Material and Methods

### 2.1. List of Primers and siRNA

Primers and siRNA sequence detailed in Table 1.

### 2.2. Plasmids and siRNA

The HCV consensus clone used was derived from a Japanese patient with fulminant hepatitis and has been designated pJFH-1 (genotype 2a), which was kindly provided by Dr. Takija Wakita [29]. HCV Stem loop DNAs were cloned in pcDNA 3 and linearized with XbaI to make the radiolabeled probe and RNA. siPSPC1 was used from IDT for silencing PSPC1. Recombinant PSPC1 protein was expressed using clone pET28a-PSPC1, cloned between EcoRI and BamH1. A non-specific siRNA (siNsp) (Dharmacon, D-001810-01-05) was used in the experiments as control.

### 2.3. Cell Lines and Transfections

Huh7.5 cells were provided by the laboratory of Prof. Charles M. Rice, Rockefeller University, Apath, LLC (New York, NY, USA), and maintained in Dulbecco’s modified Eagle’s medium (Gibco, DMEM, high glucose, pyruvate; 11995073) supplemented with 100 U/mL of penicillin, 100 mg/mL of streptomycin (HiMedia, A004), and non-essential amino acids (Gibco) with 10% fetal bovine serum (Gibco, 10270106). Liposome-mediated transfection was performed for transfection of in-vitro-transcribed HCV-JFH1 RNA according to the manufacturer’s protocol. Briefly, HCV-JFH1 RNA was transfected in Huh7.5 cells using Lipofectamine 2000 (Invitrogen, 11668019) at an RNA/lipofectamine ratio of 1:2. HCV JFH1 RNA or siRNAs were transfected in cells maintained in OptiMEM (Invitrogen) using Lipofectamine-2000 (Invitrogen) according to manufacturer’s protocol. Six hours post-transfection, OptiMEM was replaced by DMEM containing 10% serum. Cells were harvested at different time points post-transfection by RIPA lysis buffer or TRI reagent (Sigma, T9424) for protein and RNA isolation, respectively.

### 2.4. In Vitro Transcription

To make the template for in vitro transcription, the pJFH1 plasmids were digested by Xba I (NEB), HCV 5’Luc3’, and 3’Luc with EcoR1 and purified by phenol–chloroform (1:1), and DNA was precipitated at −20 °C with 2.5 volumes of absolute ethanol and sodium acetate, pH 5.2 (1/10th volume). JFH1 full-length DNA was transcribed in vitro via a T7 polymerase (Promega, P1300) kit using the manufacturer’s protocol in the presence of RiboLock RNase inhibitor (Thermo Scientific, EO0381, Waltham, MA, USA); then, followed by DNase I (Promega, P1300) digestion was performed. RNA was purified by phenol–chloroform followed by isopropanol precipitation at RT, 12,000 rpm, for 20 min, and 70% ethanol wash at RT, 12,000 rpm, for 10 min. RNA was quantified using a nanospectrophotometer. To make HCV RNA 5’ and 3’ UTRs RNA, DNA was transcribed by T7 polymerase (Thermo, EP0111) in the presence of RiboLock RNase inhibitor (Thermo Scientific, EO0381), rNTPs (Promega, P1132, P1142, P1152, P1162) at 37 °C for 2 h, followed by phenol–chloroform, and precipitated by absolute ethanol (2.5 volumes) and sodium acetate, pH 5.2 (1/10th volume). 

### 2.5. Total RNA Isolation, cDNA Preparation, and Semiquantitative PCR

Total RNA was isolated with TRI Reagent^TM^ (Sigma) from cells according to the manufacturer’s protocol. Briefly, TRI reagent was added onto the cell pallet and mixed via vortexing. To 1/5th volume of TRI reagent, chloroform was added, followed by vortexing. The solution was kept at RT for 10 min followed by spinning at 12,000 rpm, 4 °C, for 20 min. The supernatant was collected and transferred into new tubes, and an equal volume of isopropanol was added and mixed via vortex and precipitated at 12,000 rpm, 4 °C, for 30 min. The isolated RNA was treated with 3–5 units of DNase I (Thermo Scientific, EN0521) at 37 °C for 45 min, purified with acidic phenol–chloroform, and precipitated overnight with 1/10th 3M sodium acetate (pH 5.2) and 2.5 volumes of 100% ethanol. A total of 600 ng of RNA were used to make cDNA using specific reverse primers, control reverse primers, and M-MuLV RT (RevertAid, Thermo Scientific, EP0441) in the presence of an RNase inhibitor at 42 °C for 1 h and 75 °C for 10 min according to the manufacturer’s protocol. Semiquantitative PCR was performed using Taq polymerase (Thermo), dNTPs, and gene-specific forward and reverse primer. The reaction was carried out at 95 °C for 5 min, 35 cycles at 95 °C for 30 s, 55 °C for 30 s, and 72 °C for 30 s in a thermal cycler (Bio-Rad, San Francisco, CA, USA), and the reaction mixture was run on agarose gel and visualized in Gel Doc (Bio-Rad, San Francisco, CA, USA).

### 2.6. Quantitative Reverse Transcription PCR (qRT-PCR) 

Real-time quantification for mRNAs was performed via the SYBR green method (DyNAmo Flash SYBR green qPCR kit^TM^, Thermo Scientific). The thermocycling conditions for SYBR green assay system were as follows: 1 cycle at 95 °C for 10 min, 40 cycles at 95 °C for 30 s, 55 °C for 30 s, and 72 °C for 30 s (Applied Biosystems, Foster City, California, USA). The relative gene expression changes were analyzed using the 2^−ΔΔCt^ method, where GAPDH was taken as the internal control. The 2^−ΔΔCt^ method was used to calculate the fold change. ΔCt = Ct (target gene) − Ct (endogenous control), and ΔΔCt = ΔCt (target sample) − ΔCt (control sample). After each cycle, a melting curve analysis of qPCR was performed.

### 2.7. Western Blot Analysis

Cells were lysed in RIPA buffer (20 mM Tris pH 7.5, 150 mM NaCl, 1 mM EGTA, 1% NP-40, 1% sodium deoxycholate, 2.5 mM sodium pyrophosphate, 1 mM sodium orthovanadate, and mammalian protease inhibitor) and spun at 10,000 rpm, 4 °C, for 10 min. The protein concentration was measured using Bradford reagent (Bio-Rad), and equal amounts of proteins (100 µg) were resolved on SDS-12% PAGE. Following the desired resolution, the proteins were transferred to the PVDF membrane (Merck, HVLP04700) using a semi-dry transfer apparatus (Biorad, San Francisco, CA, USA). The blot was blocked using 5% skilled milk for 1 h at room temperature. Samples were analyzed via Western blotting using different antibodies: PSPC1 (Santa Cruz, sc-374181), HRP-tagged actin (Sigma Aldrich, A3854), and hepatitis C virus core 1b antibody (Abcam, C7-50), followed by HRP-conjugated secondary anti-mouse and anti-rabbit antibodies. Protein antibody complexes were analyzed via chemiluminescence using Immobilon Western systems (Biorad, San Francisco, CA, USA) in ChemiDoc (GE healthcare, Chicago, IL, USA). The densitometry of the Western blotting images was conducted using Muli Guage version 2.3. 

### 2.8. Immunofluorescence 

For immunofluorescence staining, ~0.1×10^6^ Huh7.5 cells were seeded on coverslips in a 24-well plate for 14 h, followed by transfection of HCV-JFH1 RNA. Post-transfection, cells were washed twice with cold 1× phosphate-buffered saline (PBS) and fixed using 4% paraformaldehyde at room temperature (RT) for 20 min. After fixing, the cells were again washed twice with 1X PBS. Permeabilization was conducted via 0.1% Triton X-100 for 4 min at room temperature; cells were incubated with 3% bovine serum albumin (BSA) PBS at RT for 1 h. They were then incubated with the primary antibodies for 4 h at RT and then further incubated with Alexa-488-conjugated anti-rabbit secondary antibodies and Alexa-633-conjugated anti-mouse antibodies for 30 min at RT in the dark. After washing with PBS, DAPI was added onto coverslips for 10 min then mounted into glass slides. Images were taken using a Zeiss confocal microscope 710, and image analysis was conducted using ZEN Black software 2.3.

### 2.9. Recombinant PSPC1 Protein Purification

Recombinant PSPC1 proteins were prepared in *Escherichia coli* Rosetta cells transformed with the pET28a-PSPC1 vector. The expression of recombinant PSPC1 was induced with 0.5 mM isopropyl-1-thio-β-d-galactopyranoside (IPTG, Sigma, I6758) at an optical density of 0.8–1.0 OD at 660 nm and grown overnight at 25 °C at 180 RPM in an incubator shaker. The cells were pelleted, resuspended in lysis buffer (50 mM Tris pH 7.5, 300 mM NaCl, and 10% glycerol), PMSF, and bacterial protease inhibitor (Sigma, P8465), and disrupted on ice via sonication. All subsequent steps were carried out at 4 ºC. The lysates were cleared via centrifugation at 10,000 rpm for 30 min; meanwhile, Ni-NTA-agarose beads were washed thrice with lysis buffer at 1000 rpm and 4 °C for 2 min. The supernatant was incubated with Ni-NTA-agarose slurry (Qiagen, 30210) while rocking for 4 h. The lysate was loaded onto a column, and the flow-through was discarded. The column was washed with 80–100 mL of wash buffer (50 mM Tris pH 7.5, 300 mM NaCl, and 40 mM imidazole). The bound protein was eluted with 200 µL of 250 and 500 mM of an elution buffer containing imidazole. Purified fractions were pooled and processed for dialysis. The eluted proteins were dialyzed twice at 4 °C for 4 h of dialysis buffer (50 mM Tris, pH 7.4, 100 mM KCl, 7 mM β-mercaptoethanol (β-ME), and 10% glycerol), aliquoted, flash frozen, and stored in a −80 °C freezer. The concentration was checked via SDS-PAGE using a standard BSA concentration followed by silver staining. 

### 2.10. Radiolabeled Probe Synthesis

RNA was transcribed in vitro using linearized constructs under the T7 promoter. pcDNA3 constructs containing the HCV UTRs were digested with EcoR1 (NEB, R3101, respectively, and used as a template for RNA synthesis using T7 RNA polymerase (Thermo scientific, EP0111) and α-^32^P UTP. The transcription reaction was carried out under standard conditions at 37 °C for 1.5 hr. After ammonium acetate and alcohol precipitation, RNA was resuspended in nuclease-free water. For radiolabeled RNA, 1 µL of the prepared RNA probe was spotted onto DE81 filter paper (Whatman) and washed with phosphate buffer and 100% ethanol, and the incorporated radioactivity was measured using a scintillation counter.

### 2.11. UV-Induced Cross-Linking 

UV-induced cross-linking was carried out as described earlier [30]. Briefly, α-^32^P RNA probes were allowed to form complexes with recombinant proteins in 1X RNA-binding buffer (5 mM HEPES pH 7.6, 25 mM KCl, 2 mM MgCl2, 3.8% Glycerol, 2 mM DTT, and 0.1 mM EDTA) at 30 °C for 30 min and were then UV-irradiated for 20 min. The mixture was treated with 30 μg of RNase A (Sigma, R6148), separated on gradient SDS-polyacrylamide gel (SDS-PAGE), and analyzed via phosphor imaging. The densitometry of the Western blotting images was conducted using Muli Guage version 2.3. For competitive UV-crosslinking experiments with cold RNA, the indicated molar excess of unlabeled RNAs with a labeled RNA probe were added to the reaction mixture. For the competitive UV-crosslinking experiments with two recombinant proteins (RPS5 and PSPC1), the recombinant proteins were added in equal ratio of their molecular mass at 1X followed by increased competition in equal ratio of their mass.

### 2.12. Immunoprecipitation—Reverse Transcription (IP-RT)

To assess the association of PSPC1 with HCV RNA, IP-RT was performed. Day 1 protein G beads were washed three times in polysome lysis buffer (PLB buffer) and pre-blocked with 0.4% BSA-PLB for 1 h at RT. This was followed by a wash with PLB buffer and then incubation with the anti-PSPC1, anti-HuR, and control IgG antibodies in polysome lysis buffer for 16 h at 4 °C. For siPSPC1, followed by IP-RT, the beads were incubated with anti-PSPC1 and anti-RPS5 antibody. On day 2, whole-cell lysates were prepared in polysome lysis buffer in the presence of mammalian protease inhibitor (Sigma, P8340), RNase inhibitor (Thermo Scientific), and lysate, rocking for 45 min, followed by centrifugation at 12,000 rpm, 4 °C, for 15 min. The supernatant was transferred into new tubes and kept for pre-clearing with protein G beads for 1 h at 4 °C while rocking. Cell lysate was used for binding. The lysate was incubated with the desired antibody overnight at 4 °C, and 10% of input RNA and protein was collected. On day 3, the lysate was washed thrice with PLB buffer, and protein and RNA were prepared for Western blotting and RT-PCR. Protein lysate was prepared by adding SDS dye and heating for 5 min and used for Western blotting. RNA sample lysate was treated with 0.1% SDS and 3 μg proteinase K at 50 °C for 30 min, followed by RNA isolation and RT-PCR to detect the presence of HCV RNA for siPSPC1, followed by IP-RT. These data were normalized with the RNA pulldown in the IgG fraction, which was further normalized with the input RNA, both for siNsp and siPSPC1 fraction, to show the overall increase or decrease in the association of viral RNA to RPS5 or PSPC1 upon the silencing of PSPC1.

### 2.13. Polysome Profiling 

Huh7.5 cells were transfected with siNsp or siPSPC1 RNA. After 16 h of transfection, they were transfected with HCV-JFH1 RNA. At 48 h post-transfection, the cells were treated with cycloheximide (100 µg/mL) for 10 min at 37 °C. Subsequently, the cells were washed with ice-cold phosphate-buffered saline (PBS) containing cycloheximide, followed by a wash with hypotonic buffer [5 mM Tris-HCl (pH 7.5), 1.5 mM KCl, 5 mM MgCl2, and 100 µg/mL cycloheximide]. Cells were then scraped in ice-cold lysis buffer [5 mM Tris-HCl (pH 7.5), 1.5 mM KCl, 5 mM MgCl2, 100 µg/mL cycloheximide, 1 mM dithiothreitol (DTT), 200 U/mL RNasin, 200 µg/mL tRNA, 0.5% Triton X-100, 0.5% sodium deoxycholate, and 1X protease inhibitor cocktail] and incubated on ice for 15 min. Following incubation, a final concentration of 150 mM of potassium chloride (KCl) was added to the lysate. After centrifuging the lysate at 3000× *g* for 8 min at 4 °C, the supernatant was collected and can either be processed right away or flash-frozen and kept for later use at −80 °C. Then, 1.0–1.5 μg of total protein was loaded onto a 10–50% sucrose gradient and centrifuged at 36,000 rpm for 2 h at 4 °C in SW41 rotor (Beckman, California, USA). A polysome profiler (BioComp, New Brunswick, Canada) was used to visualize polysome profiles and collect fractions. Individual fractions were then used to separate monosomes and polysomes, and RNA was isolated for RT-PCR. The final data were normalized with a housekeeping gene GAPDH and further normalized with the amount of input RNA present in each condition to nullify the possible effect of an overall increase in polysomes due to the silencing of PSPC1.

### 2.14. Statistical Analysis

The data were expressed as mean ± SD. Statistical significance was calculated using a two-sided Student’s *t*-test. The criteria for statistical significance were *p* ≤ 0.05 (*) or *p* ≤ 0.01 (**) or *p* ≤ 0.001 (***) or unless specifically mentioned in figure legends.

## 3. Results

### 3.1. PSPC1 Is Relocalized to the Cytoplasm and Negatively Regulates HCV RNA Translation and Replication

**Figure 1:**
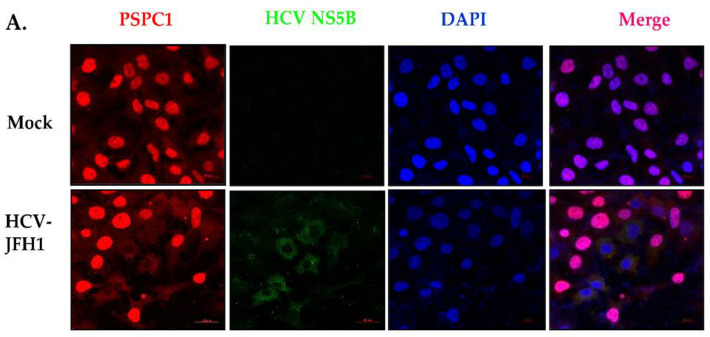

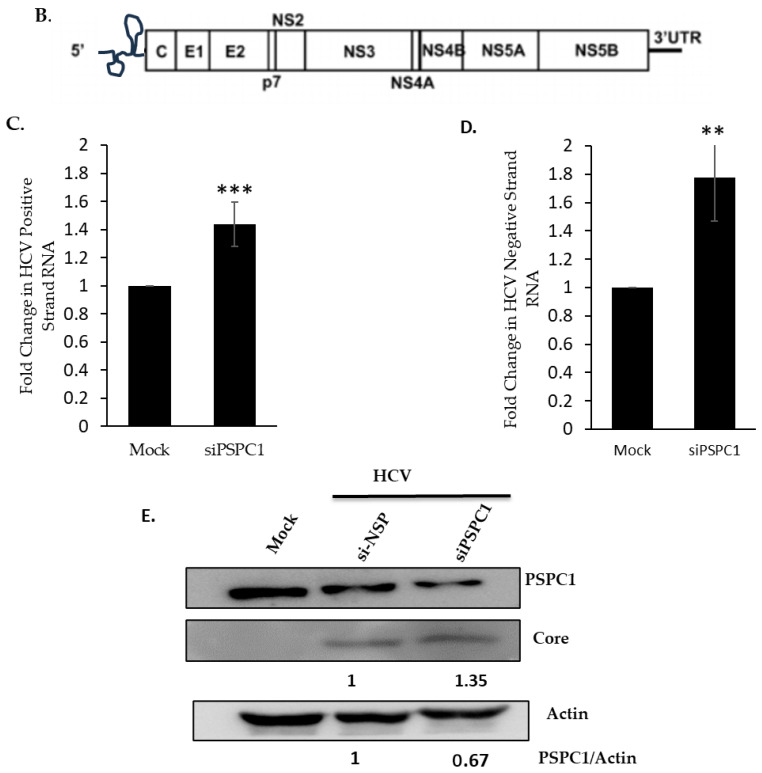
Localization of PSPC1 and its effect on HCV-JFH1 RNA transfection. (**A**) Huh7.5 cells were transfected with HCV-JFH1 RNA followed by immunofluorescence. Alexa Fluor conjugated secondary antibodies against PSPC1 (Red) and NS5B (Green) were used for detection. The nucleus was counterstained with DAPI. (**B**) A representative image of HCV JFH1 RNA (adapted from Matsui et al. J Virol. 2012) [31]. (**C**) Huh7.5 cells were transfected with siNsp and siPSPC1, and after 24 h transfection, HCV-JFH1 RNA was transfected; at 48 h post-transfection, cells were harvested for RNA isolation and Western blotting. A positive strand of HCV RNA was detected via qRT-PCR upon siPSPC1. (**D**) A negative strand of HCV RNA was detected upon siPSPC1 treatment using qRT-PCR. (**E**) Western blot analysis showing the effect of the silencing of PSPC1 (** *p* < 0.01; *** *p* < 0.001).

PSPC1 is known to relocalize to the cytoplasm under different cellular stress conditions. However, it is known to exist primarily in the nucleus. To elucidate the role of PSPC1 in the HCV life cycle, we checked whether PSPC1’s subcellular localization changes in response to HCV infection. Huh7.5 cells were infected with HCV-JFH1 RNA, and at 48 h post-transfection, the cells were immuno-stained with anti-PSPC1 and anti-NS5B antibodies. PSPC1 was found to be in the cytoplasm after HCV-JFH1 RNA transfection (Figure 1A). We also calculated the co-localization coefficient of NS5B with PSPC1 and observed no colocalization. Further, to study the role of PSPC1 in the HCV life cycle, partial silencing of PSPC1 was conducted in Huh7.5 cells, followed by HCV-JFH1 transfection for 48 h. The level of HCV-core protein was taken as a measure of change in viral RNA translation, and the positive- and negative-sense HCV RNA was examined using quantitative reverse transcription-PCR (qRT-PCR) (Figure 1B–E). Partial silencing of PSPC1 led to an increased level of viral translation and replication, thereby negatively regulating the virus life cycle.

### 3.2. PSPC1 Specifically Interacts with HCV 5’UTR

**Figure 2:**
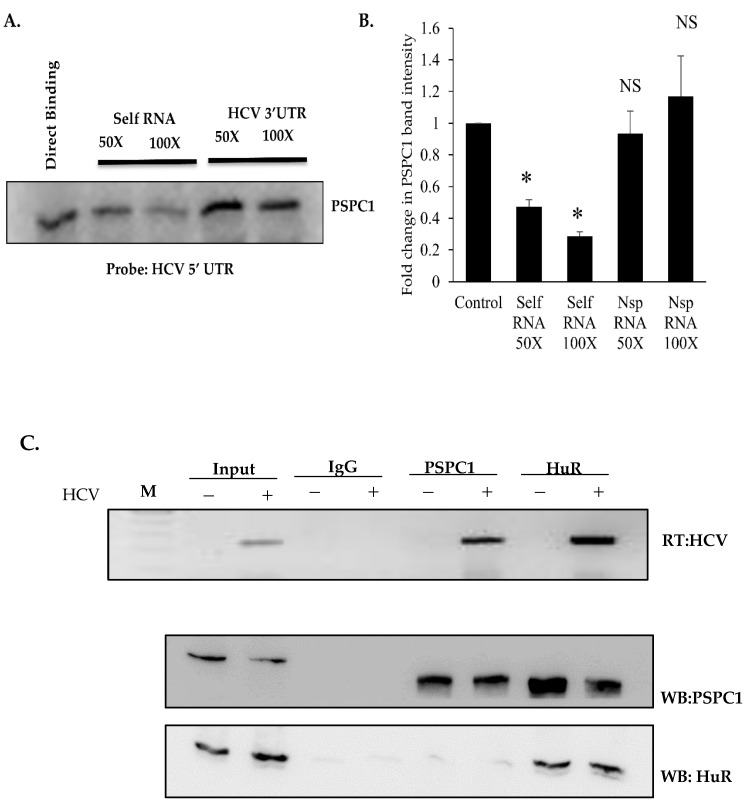
Binding of PSPC1 with HCV RNA. (**A**) UV cross-linking assay using recombinant PSPC1 and radio-labeled 5’ UTR RNA. Lane 1 corresponds to the interaction between RNA and recombinant PSPC1 protein. For competition, 50- and 100-fold molar excesses of unlabeled 5’ UTR RNA (lanes 2 and 3) or HCV 3’ UTR as non-specific RNA (lanes 4 and 5) were also included in the reaction. (**B**) Densitometry of the competition UV-crosslinking. (**C**) IP-RT assay was performed by incubating protein G beads with IgG, PSPC1, and HuR antibodies, followed by incubation with Huh7.5 cell lysate. Further, the complex was processed for RNA isolation and Western blotting. HCV RNA levels were detected using HCV-specific primer in semi-quantitative PCR, and Western blotting was performed to check the pulldown by anti-PSPC1 and anti-HuR antibodies. (P = NS, not significant; * *p* < 0.05).

To check the direct interaction of PSPC1 with HCV 5’UTR, a competition UV-crosslinking experiment was conducted whereby recombinant PSPC1 was incubated with α-^32^P-labeled HCV 5’ UTR, followed by UV-crosslinking and RNase A treatment. The complexes were resolved on SDS-12% PAGE and detected via phosphor imaging. The results showed that PSPC1 could bind specifically to HCV 5’ UTR but not 3’ UTR (Figure 2A,B). To check the interaction of PSPC1 with HCV viral RNA upon infection, HCV-JFH1 RNA was transfected into Huh7.5 cells for 48 h, followed by immunoprecipitation with anti-PSPC1 antibody along with anti-HuR antibody as a positive and IgG as a negative control, respectively. The RNA isolated from the immunoprecipitated RNP complexes were checked for HCV RNA using semi-quantitative RT-PCR; the results showed that HCV RNA interacts with PSPC1 protein upon HCV-JFH1 RNA transfection (Figure 2C).

### 3.3. PSPC1 Interacts with SLIV Domain of HCV 5’UTR and Competes with RPS5

**Figure 3:**
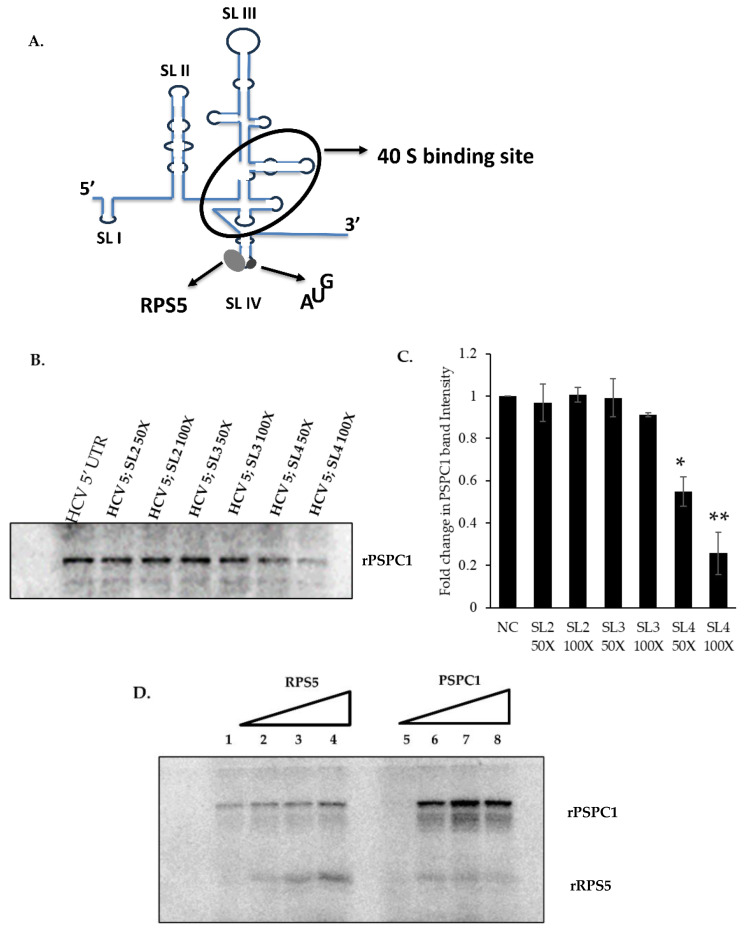

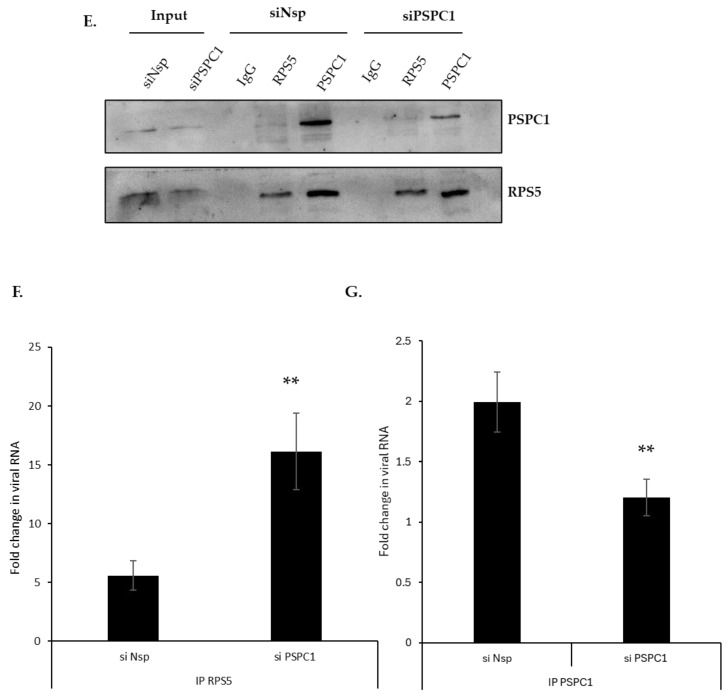
Binding of PSPC1 with different stem loops of HCV 5’ UTR and competition with RPS5. (**A**) A schematic representation of the HCV IRES showing interaction with RPS5 and ribosome loading. (**B**) UV-crosslinking assay with α-^32^P-labeled full-length 5’UTR of HCV RNA alone or in the presence of the indicated molar excess (50× and 100×) of unlabeled stem–loop RNAs of HCV 5’ UTR. (**C**) Densitometry of UV crosslinking experiment from (B) represented in graphical format. (**D**) Competition UV cross-linking of HCV IRES and PSPC1 in the presence of rRPS5 (lane 1–4). Competition UV cross-linking of HCV IRES and RPS5 in the presence of rPSPC1 (lane 5–8). (**E**) Western blot showing PSPC1 and RPS5 protein after HCV-JFH1 RNA transfection in Huh7.5 cells, followed by immunoprecipitation using anti-PSPC1 antibody, anti-RPS5 antibody, or IgG isotype antibody (as a negative control) in the presence of siPSPC1 or siNsp. (**F**) qRT-PCR data showing fold change in viral RNA upon immune-precipitation with anti-RPS5 antibody. (**G**) Fold change in viral RNA using qRT-PCR upon immune precipitation with anti-PSPC1 antibody. (* *p* ≤ 0.05, ** *p* < 0.01).

HCV IRES consists of several stem–loop (SL) domains. The SL IV is an important region of the IRES, which includes the AUG start codon and the RPS5 (ribosomal protein S5) binding site required for polypeptide synthesis (Figure 3A). To identify the binding domain of PSPC1 in the IRES region, a competition UV-crosslinking experiment was performed whereby recombinant PSPC1 was incubated with α-^32^P-labeled HCV 5’ UTR and unlabeled RNA of different domains of the IRES, followed by UV-crosslinking and RNase A treatment. The complexes were resolved on SDS-12% PAGE and detected via phosphor imaging; the results showed that PSPC1 specifically interacts with the SLIV region of the IRES (Figure 3B, C). To identify whether the interaction of PSPC1 with SLIV can affect the binding of RPS5 protein, competition UV crosslinking was conducted with α-^32^P-labeled SLIV along with increasing concentrations of RPS5 and PSPC1, respectively. The results showed that PSPC1 can compete and remove RPS5 from SLIV but RPS5 cannot remove PSPC1 from the SLIV region, which may be due to the higher affinity of PSPC1 for the SLIV region as compared to RPS5 protein (Figure 3D). RPS5 is a very important ribosomal protein previously shown to play an important role in ribosomal loading at the HCV IRES via its interaction with the SLIV region. To investigate whether this interaction is affected by the presence of PSPC1, immunoprecipitation via anti-RPS5 and anti-PSPC1 antibodies followed by RTPCR upon HCV-JFH1 transfection was performed in the background of the silencing of PSPC1. The results showed that the association of RPS5 with the viral RNA increased upon siPSPC1 at 48 h post-JFH1 transfection upon normalization of the viral RNA immunoprecipitated with IgG as a negative control (Figure 3E–G). 

### 3.4. PSPC1 Competes with RPS5 Protein Binding at the SLIV Region and Affects the Ribosome Loading

**Figure 4:**
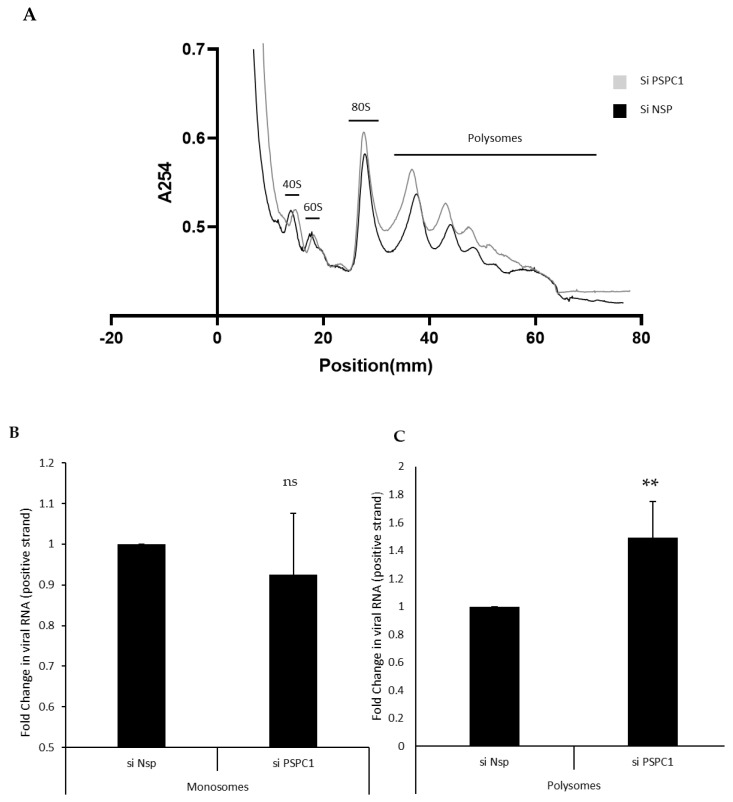
Effect of partial silencing of PSPC1 in ribosome loading on HCV RNA. (**A**) Polysome profiling of JFH1 RNA transfected cells upon of siNsp and siPSPC1 transfections conditions using sucrose density centrifugation. (**B**,**C**) Fold change in the viral RNA in monosomes and polysomes upon the silencing of PSPC1. (P = NS, not significant; ** *p* < 0.05).

To check the role of PSPC1 in ribosome loading onto HCV RNA, polysome profiling of JFH1 RNA transfected cells was conducted in the background of the silencing of PSPC1. The overall polysomal profile did not change upon siPSPC1 conditioning (Figure 4A), but there was an increase in the HCV RNA in the polysomal fraction (Figure 4B,C). Results show that PSPC1 interacts with the SLIV region of the HCV 5’UTR and affects ribosomal loading on the viral RNA by competing with RPS5 protein. 

### 3.5. HCV-JFH1 Transfection Decreases PSPC1 Protein Level

**Figure 5:**
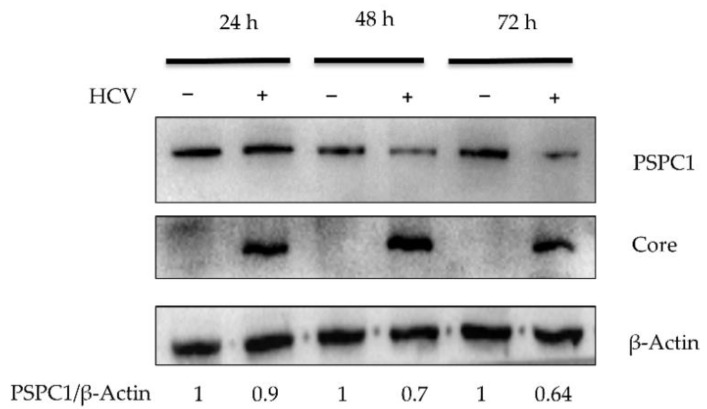
PSPC1 level upon HCV-JFH1 RNA transfection. Huh7.5 cells were transfected with HCV-JFH1 RNA, followed by cells harvested at different time points and processed for Western blotting. PSPC1 protein levels were detected at 24 h, 48 h, and 72 h post-transfection.

PSPC1 was shown to negatively regulate HCV translation. To check the effect of HCV infection, its protein level was checked upon HCV-JFH1 transfection in Huh7.5 cells at different time points. The results showed that PSPC1 protein levels gradually decrease upon HCV-JFH1 RNA transfection (Figure 5). It is possible that PSPC1 reduces HCV RNA translation and virus proliferation as part of host defense and the virus decreases the PSPC1 protein level as a viral strategy. 

## 4. Discussion

Hepatitis C virus (HCV) infection remains a significant global health concern, with a need for further understanding of the molecular mechanisms involved in its life cycle. Several cellular proteins have been discovered that are linked to HCV infection. However, only a few are known to interact with HCV RNA [5,7,11,12]. In the present study, we demonstrate that PSPC1 relocalizes to the cytoplasm, where it binds to HCV RNA and causes a reduction in HCV RNA translation. Based on these findings, we show that PSPC1 negatively regulates the HCV life cycle. 

The first significant finding of this research is the relocalization of PSPC1 to the cytoplasm upon HCV-JFH1 infection. This translocation suggests a potential shift in PSPC1 function during viral infection, as it primarily resides in the nucleus under normal conditions. PSPC1 has been known to move to the cytoplasm in response to a variety of cellular stresses; therefore, this translocation is expected during viral infection. However, its specific relocalization during HCV infection emphasizes its probable involvement in the viral life cycle. We have reported earlier how HCV protein-mediated kinase regulation can influence the HuR relocalization which, in turn, regulates the HCV life cycle [14]. Similarly, it would be interesting to investigate the molecular mechanism of the relocation of the PSPC1. Interestingly, we observed that PSPC1 negatively regulates the HCV translation, whereas PSPC1 is reported to enhance the HDV life cycle [27]. These distinct roles of PCPC1 in regulating viral replication emphasize the complexity of host–virus interactions. Whether specific post-translational modification is required for these dual roles of PSPC1 needs to be further investigated.

UV-crosslinking experiments showed that PSPC1 binds specifically to HCV IRES RNA, a critical region for viral translation. This interaction was confirmed in infected cells using IP-RT experiments, suggesting its relevance in the context of HCV infection. Together, these results elucidate a previously unknown aspect of HCV–host interactions and reveal the RNA binding property of the host nuclear protein that controls the HCV life cycle. 

Moreover, the study identified the SLIV domain of the HCV 5’ UTR as the specific binding site for PSPC1. PSPC1’s specific binding to the SLIV region of the HCV IRES resulted in competition for the binding of RPS5 protein to the HCV IRES, a key step for ribosomal assembly [13]. Subsequent investigation through immunoprecipitation assays revealed that the silencing of PSPC1 led to an increased association of RPS5 with viral RNA, reconfirming the fact that PSPC1’s presence disrupts the interaction between RPS5 and the SLIV region. Polysome profiling further supported these findings, demonstrating an increase in HCV RNA in the polysomal fraction upon PSPC1 silencing, indicative of enhanced ribosome recruitment. Overall, these results elucidate a novel role for PSPC1 in modulating ribosomal loading onto HCV RNA. The interplay between PCPC1 and RPS5 could be fine-tuned by the relative RNA binding affinity and protein availability or cytoplasmic abundance. 

The observed reduction in PSPC1 protein levels after HCV infection offers strong evidence for the dynamic interaction of virus proliferation strategies and host defense mechanisms. The gradual reduction in PSPC1 protein levels, as demonstrated in Figure 5, along with previous findings indicating PSPC1’s role in negatively regulating HCV RNA level, suggests a potential mechanism by which PSPC1 acts as a host defense factor against HCV infection, inhibiting viral translation. Conversely, HCV appears to counteract this host defense mechanism by diminishing PSPC1 protein levels, possibly as a viral strategy to enhance its own replication. Earlier reports showed that treatment with poly I:C enhances the formation of paraspeckles but has no effect on localization and modifies PSPC1 protein levels relatively marginally [26]. Moreover, it has been noted that PSPC1 levels remain unchanged after HSV infection [25,26,28]. PSPC1 helps in HDV replication, and active HDV replication leads to PSPC1 relocalization without concomitant changes in its level [27]. According to a different study, PSPC1 would be more stable in infected samples of the Zika virus than in mock samples [32]. Our observations in this study, in conjunction with previous findings with other viruses, indicate that PSPC1 relocalization and changes in protein level might require active HCV replication. The complex molecular race between the virus and the host is shown by this reciprocal relationship. Whether the reduction of PSPC1 protein levels upon HCV RNA transfection is due to ubiquitin or lncRNA-mediated protein degradation, transcription suppression, or regulation through miRNAs is yet to be explored [33,34,35].

## 5. Conclusions

**Figure 6:**
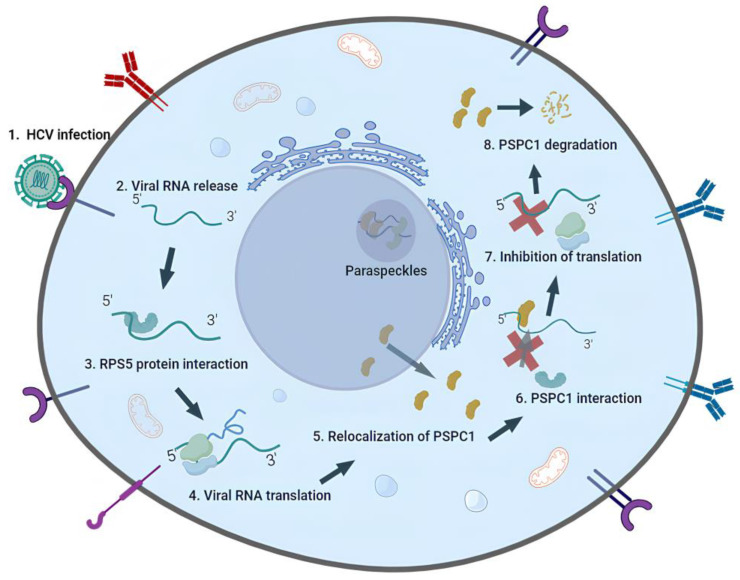
Model illustrating PSPC1, a paraspeckle protein, in the life cycle of HCV. Upon entry into the cell through receptor-mediated endocytosis, HCV undergoes uncoating, releasing its positive-strand RNA genome into the cytoplasm for translation. Translational initiation of HCV RNA occurs via its internal ribosome entry site (IRES). RPS5 is essential for locating the HCV RNA on the 40S ribosomal subunit at the start of translation. Upon HCV infection, PSPC1 relocates to cytoplasm and interacts with the 5’ UTR SLIV region of HCV IRES. As soon as it binds to the HCV IRES, it replaces the protein RPS5, which is necessary for HCV translation, which leads to a decrease in HCV RNA. It appears that PSPC1 lowers the levels of HCV RNA as a host response, and as a viral tactic, HCV lowers the level of PSPC1 protein.

Overall, the findings presented in this research shed light on the complex interplay between PSPC1 and HCV during infection. By elucidating PSPC1’s role in modulating viral RNA translation and replication, this study provides a valuable contribution to our understanding of the complex interplay between the host and the virus. This is the first study showing the involvement and possible anti-viral effect of PSPC1 during the HCV life cycle, where PSPC1 was found to relocalize to the cytoplasm and interact with the HCV IRES, affecting ribosomal loading via interaction with the SLIV region (Figure 6).

## Figures and Tables

**Table 1:** Sequence of primers and siRNA used in this study.

Name	Sequence
siPSPC1 (IDT)	5′ rUrUrCrGrUrUrCrArUrUrCrCrUrGrGrCrUrArUrCrUrAdTdT 3′
siNsp Dharmacon (D-001810-01-05)	5′ UGGUUUACAUGUCGACUAA 3′
HCV F	5′ TGCGGAACCGGTGAGTACA 3′
HCV R	5′ GAGGTTTAGGATTTGTGCTCAT 3′
GAPDH F	5′ CAGCCTCAAGATCATCAGCAAT 3′
GAPDH R	5′ GGTCATGAGTCCTTCCACGA 3′

## Data Availability

All data is available upon reasonable request.
